# Utility of pneumonia severity assessment tools for mortality prediction in healthcare-associated pneumonia: a systematic review and meta-analysis

**DOI:** 10.1038/s41598-024-63618-3

**Published:** 2024-06-05

**Authors:** Shingo Noguchi, Masahiro Katsurada, Kazuhiro Yatera, Natsuki Nakagawa, Dongjie Xu, Yosuke Fukuda, Yuichiro Shindo, Kazuyoshi Senda, Hiroki Tsukada, Makoto Miki, Hiroshi Mukae

**Affiliations:** 1 Department of Respiratory Medicine, University of Occupational and Environmental Health, Kitakyushu, Japan; 2 Department of Respiratory Medicine, Tobata General Hospital, Kitakyushu, Japan; 3 Department of Respiratory Medicine, Kita-Harima Medical Center, Ono, Japan; 4 Department of Respiratory Medicine, The University of Tokyo Hospital, Tokyo, Japan; 5 Department of Pulmonary and Respiratory Medicine, Japanese Red Cross Sendai Hospital, Sendai, Japan; 6 Division of Respiratory Medicine and Allergology, Department of Medicine, Showa University School of Medicine, Tokyo, Japan; 7 Department of Respiratory Medicine, Nagoya University Graduate School of Medicine, Nagoya, Japan; 8 Department of Pharmacy, Kinjo Gakuin University, Nagoya, Japan; 9 Department of Infection Control, The Jikei University Kashiwa Hospital, Kashiwa, Japan; 10 Unit of Translational Medicine, Department of Respiratory Medicine, Nagasaki University Graduate School of Biomedical Sciences, Nagasaki, Japan

**Keywords:** Mortality, Nursing- and healthcare-associated pneumonia, Nursing home-acquired pneumonia, Prognosis, Severity score, Bacteriology, Infectious diseases, Respiratory tract diseases

## Abstract

Accurate prognostic tools for mortality in patients with healthcare-associated pneumonia (HCAP) are needed to provide appropriate medical care, but the efficacy for mortality prediction of tools like PSI, A-DROP, I-ROAD, and CURB-65, widely used for predicting mortality in community-acquired and hospital-acquired pneumonia cases, remains controversial. In this study, we conducted a systematic review and meta-analysis using PubMed, Cochrane Library (trials), and Ichushi web database (accessed on August 22, 2022). We identified articles evaluating either PSI, A-DROP, I-ROAD, or CURB-65 and the mortality outcome in patients with HCAP, and calculated the pooled sensitivities, specificities, positive likelihood ratio (PLR), negative likelihood ratio (NLR), diagnostic odds ratio (DOR), and the summary area under the curves (AUCs) for mortality prediction. Additionally, the differences in predicting prognosis among these four assessment tools were evaluated using overall AUCs pooled from AUC values reported in included studies. Eventually, 21 articles were included and these quality assessments were evaluated by QUADAS-2. Using a cut-off value of moderate in patients with HCAP, the range of pooled sensitivity, specificity, PLR, NLR, and DOR were found to be 0.91–0.97, 0.15–0.44, 1.14–1.66, 0.18–0.33, and 3.86–9.32, respectively. Upon using a cut-off value of severe in those patients, the range of pooled sensitivity, specificity, PLR, NLR, and DOR were 0.63–0.70, 0.54–0.66, 1.50–2.03, 0.47–0.58, and 2.66–4.32, respectively. Overall AUCs were 0.70 (0.68–0.72), 0.70 (0.63–0.76), 0.68 (0.64–0.73), and 0.67 (0.63–0.71), respectively, for PSI, A-DROP, I-ROAD, and CURB-65 (*p* = 0.66). In conclusion, these severity assessment tools do not have enough ability to predict mortality in HCAP patients. Furthermore, there are no significant differences in predictive performance among these four severity assessment tools.

## Introduction

Healthcare-associated pneumonia (HCAP) is a type of pneumonia that was described in the American Thoracic Society/Infectious Diseases Society of America (ATS/IDSA) guidelines in 2005^1^. In Japan, a similar category was proposed in 2011 as nursing- and healthcare-associated pneumonia (NHCAP)^2^. Additionally, the ATS/IDSA 2019 guidelines recommended abandoning the category of HCAP and combining it with community-acquired pneumonia (CAP) to avoid unnecessary selection of extended antibiotic coverage^3^. However, the characteristics of the patients, the detection rate of multi-drug resistant pathogens, and pneumonia mortality rates are different between CAP and HCAP in some countries, including Japan^4–7^, and the concept of HCAP is still needed under the situation where there will be many elderly pneumonia patients in aging societies such as Japan. Therefore, proper prognostic tools are required to follow the appropriate clinical practice.

Severity assessment tools such as the pneumonia severity index (PSI) and CURB-65 find clinical use worldwide. In addition, tools like A-DROP and I-ROAD are widely used to predict mortality in Japan. PSI^8,9^, CURB-65^9–11^, and A-DROP^8–11^ in CAP and I-ROAD^12^ in hospital-acquired pneumonia (HAP) are shown to have good predictive efficacies for mortality. But the utility of these tools for predicting mortality in patients with HCAP has been controversial because of conflicting reports^7,13–21^. The efficacy of these tools previously reported in patients with HCAP may be primarily influenced by the participants' social backgrounds, underlying diseases, and comorbidities. Therefore, studies targeting large populations are required.

We previously conducted a systematic review and meta-analysis on the utility of PSI and CURB-65 for predicting mortality in patients with HCAP^22^. We showed that these tools lacked significant capability in HCAP, though PSI may be slightly more useful than CURB-65. However, only a few studies were included in the meta-analysis (seven for PSI and eight for CURB-65). There was insufficient data for A-DROP and I-ROAD (accessed on July 16, 2015).

In this systematic review and meta-analysis, we re-validate the significance of PSI and CURB-65 and evaluate the usefulness of A-DROP and I-ROAD for predicting mortality in patients with HCAP, aiming to reveal the effectiveness of existing severity assessment tools.

## Methods

### Search and selection criteria

This study was conducted in accordance with the Preferred Reporting Items for Systematic Reviews and the Meta-Analyses (PRISMA) statement and Meta-analysis of observational Studies in epidemiology (MOOSE) guidelines^23,24^.

We searched for studies using PubMed, Cochrane Library (trials), and Ichushi web database, and the following search words in PubMed were applied: “pneumonia [MeSH Terms]” AND (“healthcare associated pneumonia” OR “health-care-associated pneumonia” OR “healthcare-associated pneumonia” OR “nursing home acquired pneumonia” OR “nursing and healthcare associated pneumonia” OR “long term care facility” OR “extended-care facility”) AND (“severity score” OR “predict” OR “prognosis” OR “mortality score” OR “pneumonia severity index” OR “PORT score” OR “fine score” OR “A-DROP” OR “I-ROAD”) as previously reported^22^, while corresponding terms were used to search the Cochrane Library and Ichushi web database (accessed on August 22, 2022).

### Inclusion and exclusion criteria

The inclusion criteria for eligible studies were as follows: prospective or retrospective studies targeting hospitalized patients with HCAP, nursing home-acquired pneumonia (NHAP) and/or NHCAP according to the 2005 ATS/IDSA guidelines^1^ and/or the 2017 Japanese Respiratory society (JRS) guidelines^25^, evaluating severity scores of PSI^26^, A-DROP^27^, I-ROAD^12^, or CURB-65^28^ and reporting mortality outcomes and raw data for the number of patients and deaths for any item of each severity grade, written in English or Japanese as original research articles. Exclusion criteria were as follows: studies involving children; case reports, conference reports, reviews; studies including patients who did not receive inpatient treatment in hospital because of possible significant biases for the treatment contents; studies with overlapping periods at the same medical institution; and studies lacking detailed data of namely true-positive, false-positive, true-negative, and false-negative values at any severity grade for mortality.

### Data extraction and quality assessments

Two reviewers (SN and MK) independently assessed all the articles. The non-relevant studies were excluded based on the titles and abstracts after searching PubMed, Cochrane Library (trials), and Ichushi web database using the keywords, and the full texts of potentially appropriate titles and abstracts were further reviewed. The following information was collected from the included studies: geographic location, design, sample size, the mean age of participants, type of severity score, a common outcome, and mortality rate. The QUADAS-2, which includes four risk-of-bias domains and three domains of applicability^29^, was used to evaluate the risk of bias. Two investigators (SN and MK) evaluated the risk of bias using the QUADAS-2, and any disagreements were resolved by a third reviewer (NN) and discussed.

### Severity grade of PSI, A-DROP, I-ROAD, and CURB-65

The detailed calculation parameters of these four assessment tools are demonstrated in Table 1. PSI^26^ is classified into a five-class according to total score of the prognostic factors and the severity grade was categorized into ≥ IV (moderate) and V (severe) when there was a total score of 91 or more and 131 or more points, respectively. A-DROP^27^ and CURB-65^28^ is a 6-point scoring system and “more than one point” and “ more than three points” for A-DROP and “more than two points” and “more than three points” for CURB-65 was categorized into ≥ II (moderate) and ≥ III (severe), respectively. I-ROAD^12^ is classified into three grades and it was categorized into severe when three or more prognostic factors of “Predictors of life expectancy” were applied. When less than three were applied, it was categorized as moderate when one or more of the “determinants of pneumonia severity” was positive, and it was categorized as low when none of the two applied.

**Table 1:** Calculation parameters of the PSI, A-DROP, I-ROAD, and CURB-65.

Scoring system	Parameters	Points
PSI	Demographic factor	
	Age	
	Men	Age (years)
	Woman	Age (years)-10
	Nursing home resident	+ 10
	Coexisting illnesses	
	Neoplastic disease	+ 30
	Liver disease	+ 20
	Congestive heart failure	+ 10
	Cerebrovascular disease	+ 10
	Renal disease	+ 10
	Physical findings	
	Altered state of consciousness	+ 20
	Respiratory rate ≥ 30/minute	+ 20
	Systolic blood pressure < 90 mmHg	+ 20
	Body Temperature < 35 °C or ≥ 40 °C	+ 15
	Pulse rate ≥ 125/minute	+ 10
	Laboratory findings	
	Arterial blood pH < 7.35	+ 30
	Blood urea nitrogen ≥ 30 mg/dl	+ 20
	Sodium < 130 mEq/l	+ 20
	Blood glucose ≥ 250 mg/dl	+ 10
	Hematocrit < 30%	+ 10
	PaO_2_ < 60 mmHg (SpO_2_ < 90%)	+ 10
	Pleural effusion	+ 10
A-DROP	Male ≥ 70 years old, female ≥ 75 years old	+ 1
	Blood urea nitrogen ≥ 21 mg/dl or clinical dehydration	+ 1
	SpO_2_ ≤ 90% (PaO_2_ ≤ 60 mmHg)	+ 1
	Orientation disorder present	+ 1
	Blood pressure (systolic) ≤ 90 mmHg	+ 1
I-ROAD	1. Predictors of Life Expectancy	
	Malignant tumor or immunodeficiency state	+ 1
	FiO_2_ > 35% required to maintain SpO_2_ > 90%	+ 1
	Altered state of consciousness	+ 1
	Male ≥ 70 years old, female ≥ 75 years old	+ 1
	Oliguria or dehydration	+ 1
	2. Determinants of Pneumonia Severity	
	CRP ≥ 20 mg/dl	
	Chest X-ray shadow extending over 2/3 of a lung	
CURB-65	Confusion	+ 1
	Blood urea nitrogen > 20 mg/dl	+ 1
	Respiratory rate ≥ 30/minute	+ 1
	Systolic blood pressure < 90 mmHg or diastolic blood pressure ≤ 60 mmHg	+ 1
	Age ≥ 65 years old	+ 1

### Outcomes

The primary outcome in this study was short-term mortality (28-day, 30-day or in-hospital mortality).

### Statistical analysis

Paired forest plots and the pooled sensitivities, specificities, positive likelihood ratio (PLR), negative likelihood ratio (NLR) and diagnostic odds ratio (DOR) were calculated using the “*midas*” and “*metandi*” commands in the STATA 14 software (StataCorp LP, College Station, TX, USA), as previously reported^22^. In addition, the overall area under the curves (AUCs) of each severity assessment tools were calculated and compared with the Review Manager ver. 5.4 software. In eight studies^14,18,30–35^ where AUC was not described in the paper, the AUC was calculated based on the receiver operator characteristic (ROC) curves that were obtained from the raw data of the number of patients and fatalities for each severity grade using STATA 14 software. Statistical significance was set at a *p*-value of < 0.05. I^2^ statistics were used to evaluate the heterogeneity of the reported studies, as follows: 0–25%, low; 25–50%, moderate; 50–75%, high; 75–100%, very high.

## Results

### Database search and risk of bias assessment

**Figure 1:**
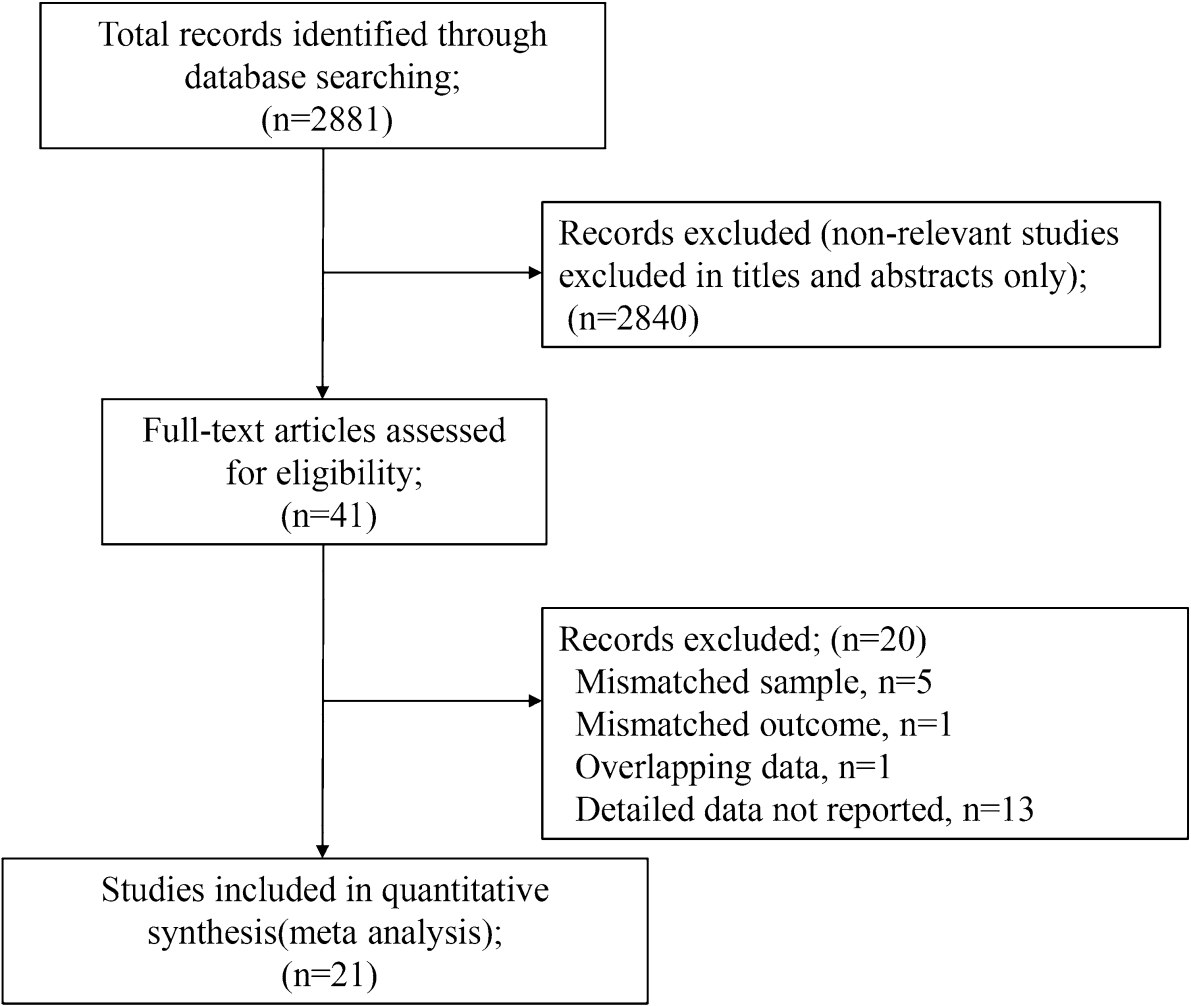
Flow chart of the study selection.

**Figure 2:**
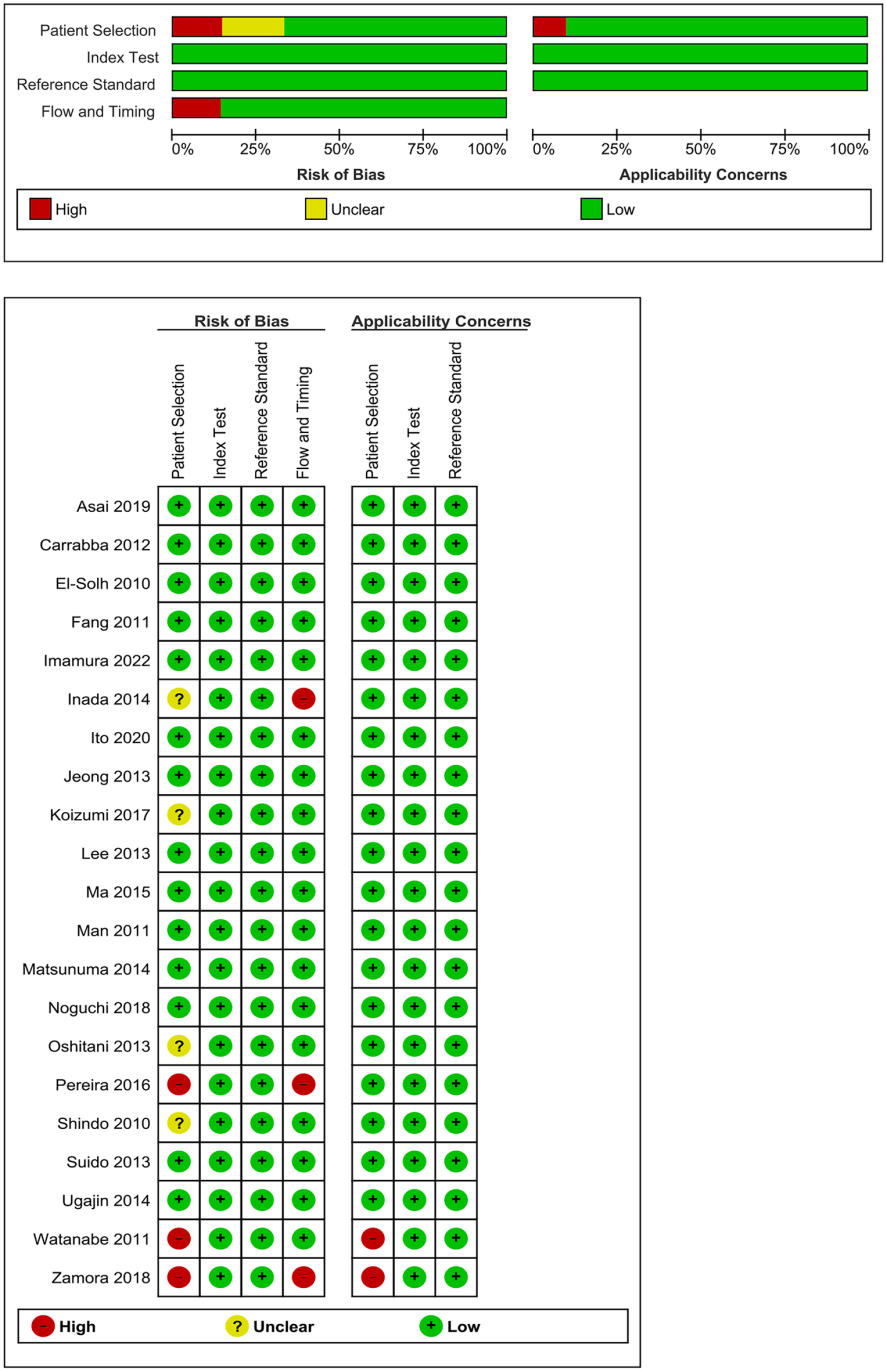
Summary of risk of bias using QUADAS-2.

A total of 2881 articles (PubMed 2276, Cochrane Library 134, and Ichushi web 471) were identified in the initial search, and 41 articles were potentially eligible after the first screening of the titles and abstracts. Next, the full text was reviewed, and 20 articles were excluded. Eventually, 21 observational studies were selected for this study (Fig. 1). The summary of the risk of bias using the QUADAS-2 in the included studies was shown in Fig. 2. For patient selection, three or two studies were evaluated as having a high risk of bias or high concern for applicability, respectively, because of the possibility of inappropriate exclusion or mismatched definition. In the patient flow and timing assessment, three studies were assessed to have a high risk of bias because of inappropriate omission or uncertainly of evaluation timing of reference standard.

### Included studies

The characteristics of the included studies are shown in Table 2. Among these 21 studies, five^6,13,18,30,36^ and 16^4,7,14,19,31–35,37–43^ were prospective and retrospective studies, respectively. The category of pneumonia was eight^7,19,31,33,36,38,39,42^, seven^13,18,32,34,35,41,43^, and six^4,6,14,30,37,40^ for HCAP, NHCAP, and NHAP, respectively. Twelve^4,7,13,14,19,30,31,36,39,41–43^ studies for PSI, 12^4,7,13,18,19,32–35,38,41,43^ for A-DROP, seven^7,13,18,19,34,41,43^ for I-ROAD, and 14^4,6,7,13,14,19,30,31,36,37,39–42^ for CURB-65 were included in this study.

**Table 2:** Characteristics of included studies.

First author, year	Country	Design	Category	Severity scores assessed	Patients number	Mean age (years)	Outcome	Mortality rate (%)
El-Solh A, 2010	USA	Retrospective	NHAP	CURB-65	457	77.4	30-day mortality	23.4
Shindo Y, 2010	Japan	Retrospective	HCAP	A-DROP	141	81.3	In-hospital mortality	21.3
Fang WF, 2011	Taiwan	Retrospective	HCAP	PSI, CURB-65	444	72.1	30-day mortality	20.9
Man SY, 2011	Hong Kong	Prospective	NHAP	PSI, CURB-65	767	83.4	30-day mortality	12.4
Watanabe M, 2011	Japan	Retrospective	HCAP	A-DROP	117	–	30-day mortality	5.1
Carrabba M, 2012	Italy	Prospective	HCAP	PSI, CURB-65	307	72.8	30-day mortality	24.1
Jeong BH, 2013	Korea	Retrospective	HCAP	PSI, CURB-65	419	67.0	30-day mortality	15.8
Lee JC, 2013	Korea	Retrospective	NHAP	PSI, CURB-65	208	80.0	30-day mortality	22.1
Oshitani Y, 2013	Japan	Retrospective	NHCAP	A-DROP	477	84.0	30-day mortality	14.0
Suido Y, 2013	Japan	Retrospective	NHCAP	A-DROP, I-ROAD	156	85.0	In-hospital mortality	32.1
Inada Y, 2014	Japan	Retrospective	NHCAP	A-DROP	215	86.0	Mortality	14.9
Matsunuma R, 2014	Japan	Retrospective	HCAP	PSI, A-DROP, I-ROAD, CURB-65	74	80.0	30-day mortality	19.0
Ugajin M, 2014	Japan	Retrospective	NHAP	PSI, A-DROP, CURB-65	138	85.0	28-day mortality	18.1
Ma HM, 2015	Hong Kong	Prospective	NHAP	CURB-65	464	85.2	30-day mortality	15.5
Pereira R, 2016	Portugal	Retrospective	NHAP	CURB-65	103	–	In-hospital mortality	46.6
Koizumi T, 2017	Japan	Retrospective	NHCAP	PSI, A-DROP, I-ROAD, CURB-65	144	81.5	30-day mortality	9.7
Murillo-Zamora E, 2018	Mexico	Retrospective	HCAP	PSI, CURB-65	109	68.2	30-day mortality	59.6
Noguchi S, 2018	Japan	Retrospective	NHCAP	PSI, A-DROP, I-ROAD	289	85.2	In-hospital mortality	6.9
Asai N, 2019	Japan	Retrospective	HCAP	PSI, A-DROP, I-ROAD, CURB-65	229	78.1	30-day mortality	7.0
Ito A, 2020	Japan	Prospective	NHCAP	PSI, A-DROP, I-ROAD, CURB-65	828	78.0	30-day mortality	12.8
Imamura Y, 2022	Japan	Prospective	NHCAP	A-DROP, I-ROAD	563	–	30-day mortality	11.9

*HCAP* healthcare-associated pneumonia, *NHAP* nursing home-acquired pneumonia, *NHCAP* nursing and healthcare-associated pneumonia.

### PSI

**Figure 3:**
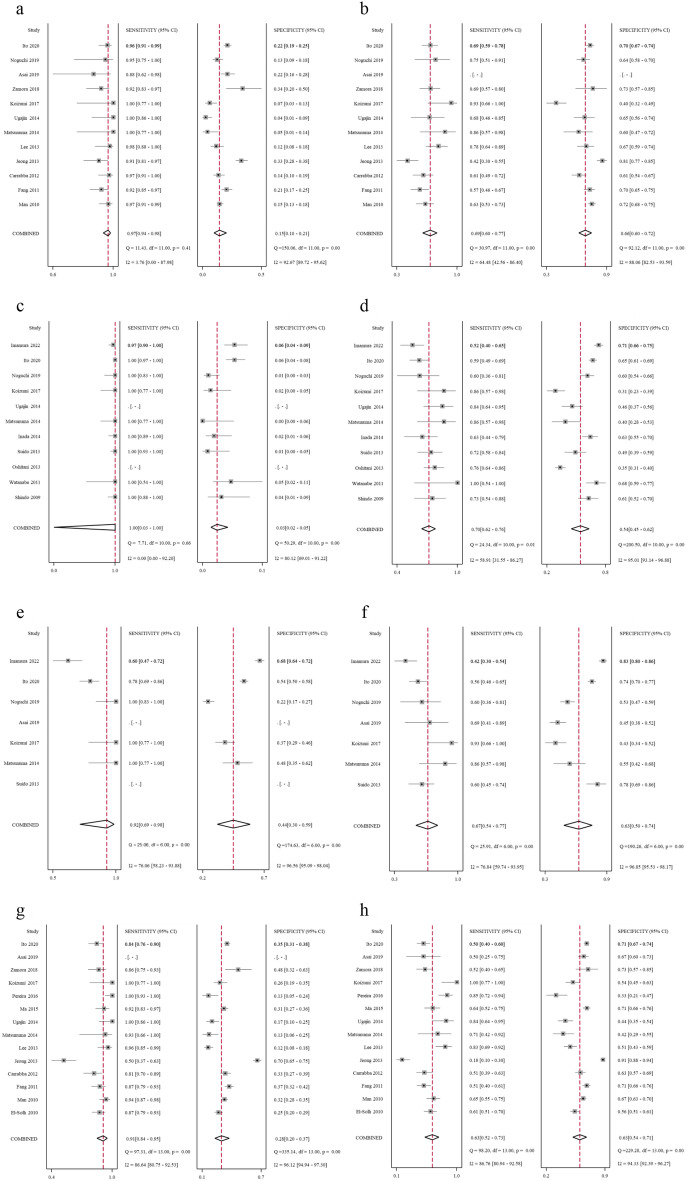
The paired forest plots of sensitivity and specificity for predicting mortality with PSI, A-DROP, I-ROAD, and CURB-65. Forest plots of sensitivity and specificity for mortality prediction with (**a**) PSI ≥ IV, (**b**) PSI V, (**c**) A-DROP ≥ I, (**d**) A-DROP ≥ III, (**e**) I-ROAD ≥ moderate, (**f**) I-ROAD ≥ severe, (**g**) CURB-65 ≥ II, and (**h**) CURB-65 ≥ III.

Twelve^4,7,13,14,19,30,31,36,39,41–43^ studies were included in the meta-analysis for the PSI score. Using a cut-off value of ≥ IV (moderate; n = 12), the pooled sensitivity, specificity, PLR, NLR, and DOR for mortality were calculated as 0.97 (0.94–0.98), 0.15 (0.10–0.21), 1.14 (1.08–1.20), 0.22 (0.12–0.38), and 5.09 (2.95–8.78), respectively (Table 3). Using a cut-off value of V (severe; n = 11), the pooled sensitivity, specificity, PLR, NLR, and DOR for mortality were 0.69 (0.60–0.77), 0.66 (0.60–0.72), 2.03 (1.82–2.27), 0.47 (0.38–0.58), and 4.32 (3.35–5.59), respectively. The forest plots and estimated sensitivities and specificities from each study are shown in Fig. 3a,b.

**Table 3:** Pooled characteristics of severity scores for predicting mortality.

	Sensitivity		Specificity		PLR		NLR		DOR	
PSI score										
≥ IV (n = 12)	0.97	(0.94–0.98)	0.15	(0.10–0.21)	1.14	(1.08–1.20)	0.22	(0.12–0.38)	5.09	(2.95–8.78)
V (n = 11)	0.69	(0.60–0.77)	0.66	(0.60–0.72)	2.03	(1.82–2.27)	0.47	(0.38–0.58)	4.32	(3.35–5.59)
A-DROP										
≥ III (n = 11)	0.7	(0.62–0.76)	0.54	(0.45–0.62)	1.50	(1.33–1.70)	0.56	(0.48–0.66)	2.66	(2.09–3.40)
I-ROAD										
≥ moderate (n = 5)	0.92	(0.69–0.98)	0.44	(0.30–0.59)	1.66	(1.39–1.98)	0.18	(0.05–0.61)	9.32	(2.86–30.3)
≥ severe (n = 7)	0.67	(0.54–0.77)	0.63	(0.50–0.74)	1.78	(1.44–2.21)	0.53	(0.42–0.68)	3.34	(2.35–4.75)
CURB-65										
≥ II (n = 13)	0.91	(0.84–0.95)	0.28	(0.20–0.37)	1.26	(1.17–1.36)	0.33	(0.23–0.46)	3.86	(2.74–5.44)
≥ III (n = 14)	0.63	(0.52–0.73)	0.63	(0.53–0.71)	1.70	(1.52–1.90)	0.58	(0.49–0.70)	2.91	(2.34–3.62)

*PLR* positive likelihood ratio, *NLR* negative likelihood ratio, *DOR* diagnostic odds ratio.

### A-DROP

Twelve^4,7,13,18,19,32–35,38,41,43^ studies were included in the meta-analysis for the A-DROP score. Using a cut-off value of ≥ III (severe; n = 11), the pooled sensitivity, specificity, PLR, NLR, and DOR for mortality were 0.70 (0.62–0.76), 0.54 (0.45–0.62), 1.50 (1.33–1.70), 0.56 (0.48–0.66), and 2.66 (2.09–3.40), respectively (Table 3). The forest plots using these cut-offs and estimated sensitivities and specificities from each study are shown in Fig. 3c,d. In one study^7^, forest plots weren’t described in Figure because the data necessary to create it in both a cut-off value of ≥ I and ≥ III was insufficient.

### I-ROAD

Seven^7,13,18,19,34,41,43^ studies were included in the meta-analysis for the I-ROAD score. Using a cut-off value of ≥ moderate (n = 5), the pooled sensitivity, specificity, PLR, NLR, and DOR for mortality were 0.92 (0.69–0.98), 0.44 (0.30–0.59), 1.66 (1.39–1.98), 0.18 (0.05–0.61), and 9.32 (2.86–30.3) respectively (Table 3). Using a cut-off value of severe (n = 7), the pooled sensitivity, specificity, PLR, NLR, and DOR for mortality were 0.67 (0.54–0.77), 0.63 (0.50–0.74), 1.78 (1.44–2.21), 0.53 (0.42–0.68), and 3.34 (2.35–4.75), respectively. The forest plots using these cut-offs and estimated sensitivities and specificities from each study are shown in Fig. 3e,f.

### CURB-65

Fourteen^4,6,7,13,14,19,30,31,36,37,39–42^ studies were included in the meta-analysis for the CURB-65. Using a cut-off value of ≥ II (moderate; n = 13), the pooled sensitivity, specificity, PLR, NLR, and DOR for mortality were 0.91 (0.84–0.95), 0.28 (0.20–0.37), 1.26 (1.17–1.36), 0.33 (0.23–0.46), and 3.86 (2.74–5.44), respectively (Table 3). Using a cut-off value of ≥ III (severe; n = 14), the pooled sensitivity, specificity, PLR, NLR, and DOR for mortality were 0.63 (0.52–0.73), 0.63 (0.53–0.71), 1.70 (1.52–1.90), 0.58 (0.49–0.70), and 2.91 (2.34–3.62), respectively. The forest plots using these cut-offs and estimated sensitivities and specificities from each study are shown in Fig. 3g,h.

### Comparisons of overall AUC among PSI, A-DROP, I-ROAD, and CURB-65

**Figure 4:**
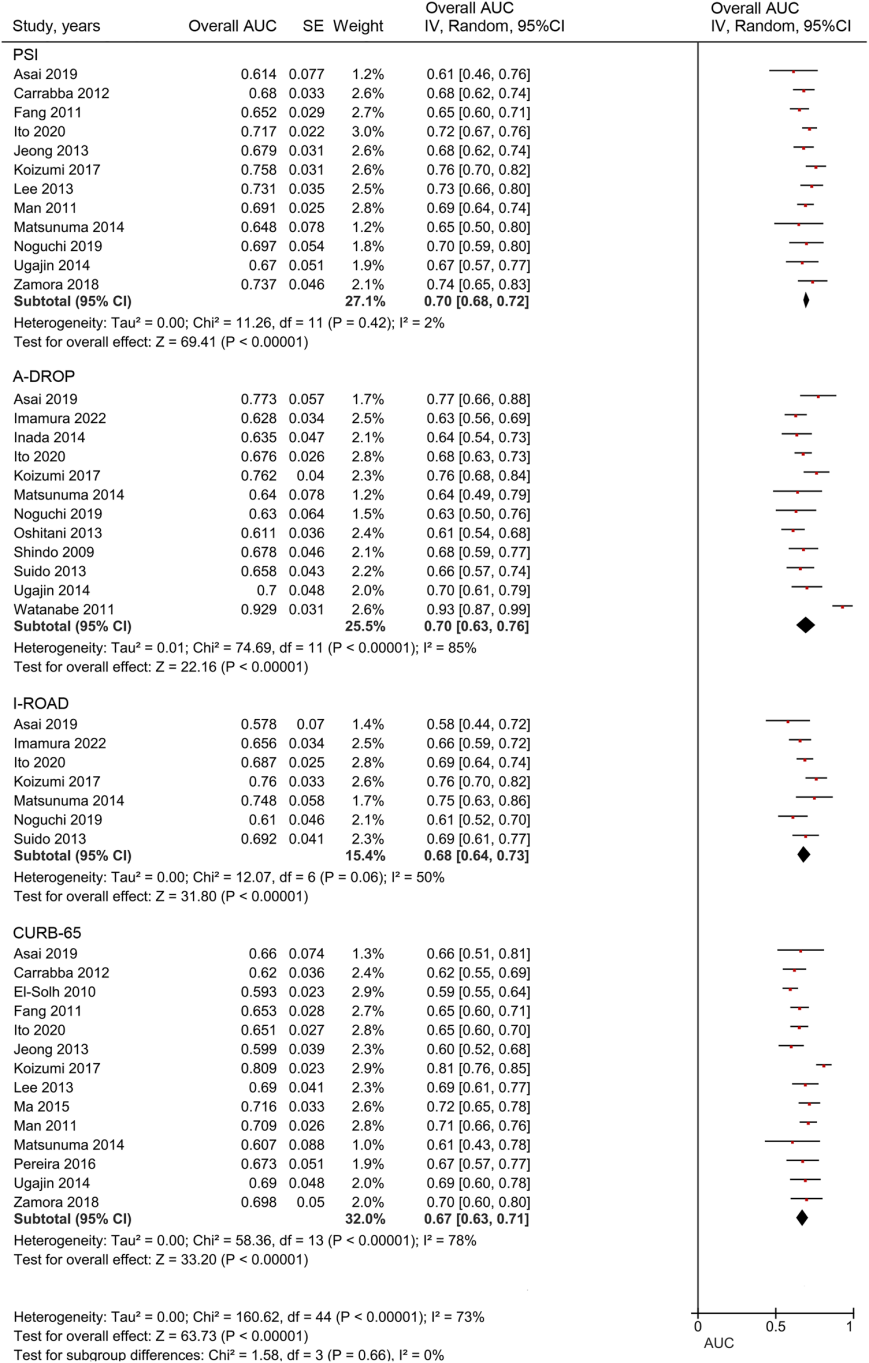
Comparison of overall AUC for PSI, A-DROP, I-ROAD, and CURB-65.

The overall AUC values were pooled from the AUC (95% CI) values reported in the included studies (Fig. 4). The overall AUCs were 0.70 (0.68–0.72), 0.70 (0.63–0.76), 0.68 (0.64–0.73), and 0.67 (0.63–0.71) for PSI, A-DROP, I-ROAD, and CURB-65 scores, respectively. No significant differences were observed (*p* = 0.66, I^2^ = 0%).

## Discussion

The present study evaluated the significance of PSI, A-DROP, I-ROAD, and CURB-65 for predicting mortality in HCAP patients. Our results indicate that these severity assessment tools cannot accurately predict mortality in patients with HCAP. In addition, there were no significant differences between these severity assessment tools.

It has been shown that PSI, A-DROP and CURB-65 in CAP and I-ROAD in HAP have high AUCs, nearly 0.8, for predicting mortality^8–12^. In this meta-analysis, the overall AUCs for these severity assessment tools for predicting mortality are 0.67–0.70, although only two reports showed high AUC values of over 0.8 for A-DROP^38^ and CURB-65^41^. AUC is often used to measure the accuracy in studies of severity assessment, and the discriminatory value based on AUC is evaluated as “poor” for 0.60–0.69, “moderate” for 0.70–0.79, “good” for 0.80–0.89, and “excellent” for 0.90–1.00, respectively^44^, although its criteria differ between studies^45^. In our study, PSI and A-DROP had “moderate” discriminative ability, while I-ROAD and CURB-65 showed “poor” discriminative ability when we follow this criteria. Overall, our results showed no significant capability for predicting mortality among the four assessment tools. Generally, patients with HCAP are highly heterogeneous, and their mortality is affected by various factors, including general conditions, laboratory data on admission to the hospital, comorbidities, antibiotic-resistant bacterial infections, and their social backgrounds, in addition, it may be also influenced by the rate of intensive care unit (ICU) admission and/or do not attempt resuscitation (DNAR); for example, the rates of ICU admission and DNAR were 0.9–26.4%^6,13,14,18,30,31,37,39^ and 24.0–55.6%^7,18,37^, respectively, although these numbers weren’t mentioned in all 21 studies. Thus, these severity assessment tools did not show enough predictive capability for mortality in HCAP patients. In addition, these results remained unchanged even when limited to NHCAP patients, although the comparison of severe grade between A-DROP and I-ROAD was only performed due to few studies evaluating PSI or CURB-65 (Supplementary Table S1 and Fig. S1).

This meta-analysis found no significant differences in overall AUCs between PSI and the remaining tools. PSI includes some comorbidities and physical and laboratory parameters as evaluation items and might be the best score for predicting mortality in the patients’ group with comorbidities such as HCAP^46^. In addition, the item “pH”, included in PSI and the SCAP score, is known as an indicator of metabolic acidosis under sepsis^31^. On the other hand, the item “age”, included in all of the severity assessment tools evaluated in this study, occupies a relatively large weight in PSI score but was not a significant risk factor for in-hospital mortality in NHCAP^5^. Further investigation is needed, but age and comorbidities may be overvalued in predicting pneumonia severity in elderly patients such as HCAP^39^. Furthermore, the influence of the general condition, such as “bedridden state” and “low serum albumin” as well as inflammatory biomarkers, such as “CRP level” and “neutrophil-to-lymphocyte ratio” has been shown for predicting mortality in elderly patients with pneumonia^47,48^. Therefore, these explain the low AUC value despite a large number of items, as the prognosis might be more strongly influenced by the ordinal general condition than the presence of comorbid diseases in these patients^43^. Similar to our results with low NLR, Chalmers et al. reported that PSI might be superior for identifying low-risk patients with low NLR (0.2 for ≥ IV and 0.5 for ≥ V) in patients with CAP^49^, although the AUC value in our results was low compared with that of CAP (0.82 for ≥ IV, 0.81 for ≥ V). Therefore, PSI may be useful for identifying low-risk patients in HCAP similar to CAP patients, and NLR below 0.1 is generally considered useful for diagnoses^50^.

A-DROP and CURB-65 are easy to use in daily clinical practice. However, these tools may not be ideal in patients with multiple comorbidities because these tools may underestimate the severity in the elderly patients with comorbidities^51^. In addition, most HCAP patients are over 65 years old, and the age index of A-DROP and CURB-65 might not be significant, although the utility of CURB, without the item “age”, was insignificant in patients with HCAP^36,37^. On the other hand, the results of this study showed that A-DROP and CURB-65 had almost similar predictive capabilities to PSI in the evaluation using overall AUC. PSI is relatively complex and often avoided in complicated environments such as an emergency room. Our results indicate that the predictive abilities of themselves were not enough to predict mortality, but A-DROP and CURB-65 can be one of the choices, instead of PSI, in clinical practice for HCAP owing to their evaluation conveniences.

Our previous study could not evaluate the utility of I-ROAD for predicting mortality in HCAP^22^ because there was only one report^19^ (accessed July 16, 2015). However, this study analyzed reports on I-ROAD published after 2015 (all Japanese studies). I-ROAD includes immunodeficiency and radiological findings, and these are a major difference from the other severity assessment tools, such as PSI, A-DROP and CURB-65. Indeed, it was reported that the prognostic ability of PSI and CURB-65 for mortality prediction in HCAP patients changed irrespective of immunosuppression^36^, consistent with our previous study^22^. In addition, radiological characteristics such as bilateral pneumonia were reported as independent risk factors for mortality in NHAP^52^. Although I-ROAD is not widely used outside Japan, it might be a viable choice in patients with HCAP since there was no significant difference between I-ROAD and other severity assessment tools although their low prognostic capability.

In addition to the major evaluation method listed above, there are various severity assessment tools such as the IDSA/ATS severity criteria^13,31^, M-ATS^30,31^, NHAP index^16^, NHAP model score^14^, qSOFA^7,43^, R-ATS rules^30^, SCAP^31,36^, SMART-COP^16,31^, SOAR^14,31,37^ and SOFA^7^, but none of them showed adequate prognostic capability. In Japan, sepsis evaluation using qSOFA and SOFA was recommended as the initial evaluation in the 2017 JRS guidelines for managing pneumonia in adults, in addition to severity assessment by PSI, A-DROP, or CURB-65^25^. Asai et al. demonstrated that SOFA scores in combination with qSOFA more accurately valuated the severity of HCAP^7^. On the other hand, it was reported that the evaluation based on clinical conditions such as malnutrition, acute mental status deterioration, health conditions requiring home care, recent hospitalization, and low BMI should be used for severity assessment^52,53^. We also showed the usefulness of combining hypoalbuminemia with the PSI or qSOFA, which increased the AUC for mortality from approximately 0.7 to 0.75 compared to PSI or qSOFA alone in NHCAP patients^43^. In addition, the efficacy of various serum biomarker such as the neutrophil to lymphocyte ratio, pro-adrenomedullin, prohormone forms of atrial natriuretic peptide, and heparin-binding protein for mortality prediction have been demonstrated in pneumonia patients^54–56^. Thus, combining new items might be needed to be considered for predicting mortality in HCAP patients.

There were some limitations in this systematic review and meta-analysis. First, the included reports had a large heterogeneity- a common drawback in meta-analyses^57^. In other word, this study had differences in each country, study design, category of pneumonia, study population, outcome, and the rates of ICU admission and DNR order, which we could not assess due to limited accessible data and a relatively small sample size. However, the heterogeneity in the HCAP population makes our findings significant. In addition, we evaluated only short-term mortality but evaluating the long-term mortality may be hoped in patient groups where a prolonged hospital stay is likely, such as HCAP cases. Second, the cut-off values of each assessment tools used for the AUC calculation may vary slightly in each report, but the cut-off values for the severity grade are generally defined in these four assessment tools and we believe that the influence on overall AUCs is therefore insignificant. Third, we could not evaluate the efficacy of A-DROP for scores more than “moderate” because many studies included in this analysis had a sensitivity of almost 100% and a specificity of 5% or less. But these results may indicate that the criteria of moderate grade in A-DROP do not have a mean for mortality prediction because most subjects, including those in the HCAP category, are adults aged 65 and above. Finally, this systematic review might have some selection bias due to the reason of limited searching database and languages included in search strategy.

In conclusion, the predictive role of PSI, A-DROP, I-ROAD, and CURB-65 for mortality was insufficient for predicting mortality in HCAP patients. We have described useful prognostic factors for mortality in HCAP patients, hoping to establish a more useful severity assessment tool with highly accurate prediction ability while considering the existing tools.

### Supplementary Information

Supplementary Information.

## Data Availability

The data that support the findings of this study are available from the corresponding authors upon reasonable request.
