# Supplementary material for: Utility of pneumonia severity assessment tools for mortality prediction in healthcare-associated pneumonia: a systematic review and meta-analysis
Source: Sci Rep. 2024 Jun 5;14:12964. doi: 10.1038/s41598-024-63618-3 (PMC11153623; doi:10.1038/s41598-024-63618-3)

**Supplementary Information**

**Title**

Utility of pneumonia severity assessment tools for mortality prediction in healthcare-associated pneumonia: a systematic review and meta-analysis

**Authors**

Shingo Noguchi, Masahiro Katsurada, Kazuhiro Yatera, Natsuki Nakagawa, Dongjie Xu, Yosuke Fukuda, Yuichiro Shindo, Kazuyoshi Senda, Hiroki Tsukada, Makoto Miki, Hiroshi Mukae

**List**

**Supplementary Table S1**.

Pooled characteristics of severity scores for predicting mortality in NHCAP patients

**Supplementary Figure S1**.

Comparison of overall AUCs for A-DROP and I-ROAD in NHCAP patients

Table S1. Pooled characteristics of severity scores for predicting mortality in NHCAP patients

|  | Sensitivity | | Specificity | | PLR | | NLR | | DOR | | AUC | |
| --- | --- | --- | --- | --- | --- | --- | --- | --- | --- | --- | --- | --- |
| A-DROP |  |  |  |  |  |  |  |  |  |  |  |  |
| ≥Ⅲ (n=7) | 0.67 | (0.58-0.75) | 0.54 | (0.42-0.64) | 1.44 | (1.24-1.69) | 0.62 | (0.52-0.73) | 2.35 | (1.78-3.10) | 0.65 | (0.61-0.69) |
| I-ROAD |  |  |  |  |  |  |  |  |  |  |  |  |
| ≥severe (n=5) | 0.63 | (0.47-0.76) | 0.68 | (0.53-0.79) | 1.93 | (1.49-2.49) | 0.55 | (0.43-0.71) | 3.48 | (2.44-4.95) | 0.7 | (0.65-0.73) |

PLR, positive likelihood ratio; NLR, negative likelihood ratio; DOR, diagnostic odds ratio; AUC, are under the curve

Figure S1. Comparison of overall AUCs for A-DROP and I-ROAD in NHCAP patients


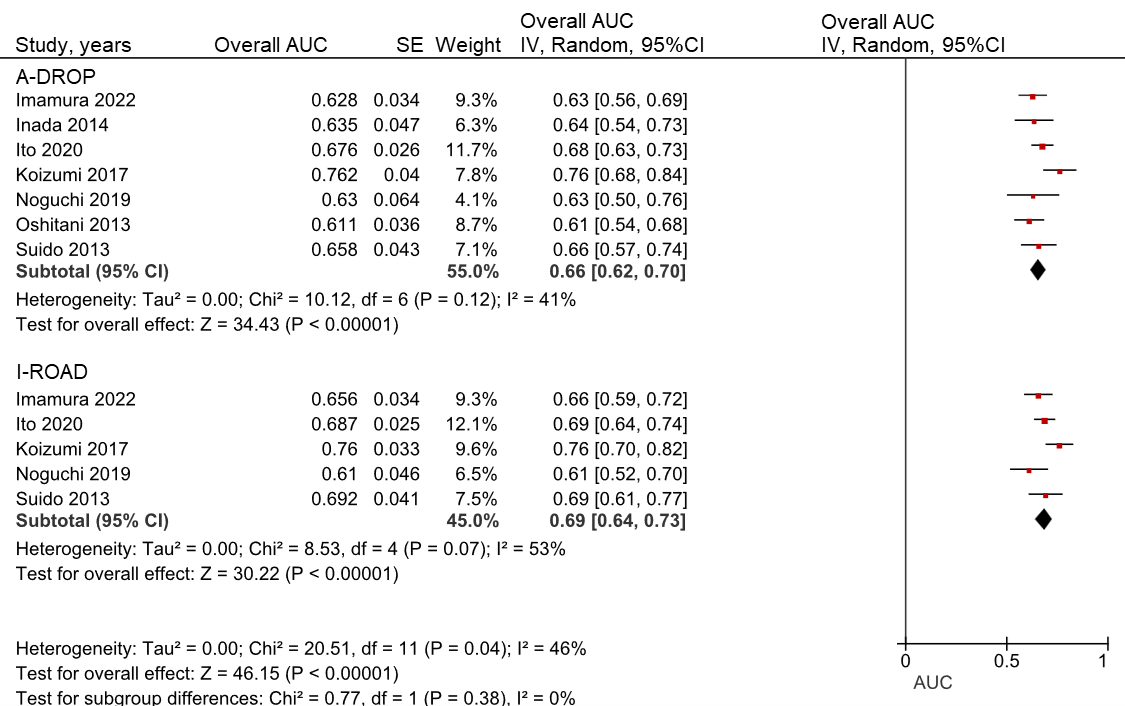

Supplement: Supplementary file 1 — Supplementary Information. [file 41598_2024_63618_MOESM1_ESM.docx]
